# Safe and effective canakinumab-treatment of neonatal onset multisystem inflammatory disease (NOMID)/ chronic infantile neurologic cutaneous and articular (CINCA)

**DOI:** 10.1186/1546-0096-13-S1-P209

**Published:** 2015-09-28

**Authors:** M Tsinti, V Dermentzoglou, E Tsitsami

**Affiliations:** 1 Pediatric Rheumatology Unit, 1st Department of Pediatrics, University of Athens, Children's Hospital “Aghia Sofia”, Medical School, Athens, Greece

## Introduction

NOMID/CINCA is the most severe phenotype of cryopyrin-associated periodic syndrome (CAPS), characterized by persistence of inflammation-mediated symptoms and overproduction of interleukin (IL)-1β, associated with significant morbidity, if untreated. In CAPS-patients early initiation of anti-IL1β-treatment appears to prevent severe disease sequelae. However, canakinumab as a 1^st^-line treatment in young infants suffering from NOMID has been scarcely reported.

## Objectives

To report the effects of early-onset canakinumab-treatment in NOMID/CINCA.

## Patients and methods

Case presentation.

## Results

A late-preterm (37-weeks- gestational-age) girl presented fever, urticarial-like rash, perilimbal redness, meningitis, elevation of WBC/neutrophils, ESR, CRP on 20 hours of life and severe anemia necessitating RBC-transfusion in the 5^th^ day of life. NOMID/CINCA was suspected on the basis of persistence of elevated inflammatory (including SAA) markers in the absence of infection-causative organisms in blood, CSF and urine, of non-responsiveness to antibiotics, of persistent CNS inflammation (CSF pleiocytosis and elevated protein) and of neutrophilic infiltration (revealed by skin biopsy) in the areas of urticarial-like rash. The detection of the c.1792A>T (p.Ile598Phe) mutation (de-novo as it was no detected in parents) in exon 3 of the *NLRP3-*gene, causative for NOMID/CINCA according to the infevers-database, confirmed the diagnosis. Brain-MRI was normal despite persistent CNS-inflammation represented by pleiocytosis and elevated protein and IL-6 and IL-8-levels in CSF (lumbar puncture performed on the 1^st^-day and 3^rd^-month of life). In peripheral blood IL-1β, IL-6 and IL-8 were undetectable. Opthalmoscopy/fundoscopy, and auditory-evoked-potentials were normal. After providing immunizations anti-IL1β-treatment with canakinumab 4mg/kg/8 weeks was initiated in the age of 4-months. Fever and rash remitted in 24h. Inflammatory markers normalized after 5-days. On 16-months of age the disease remains into remission. The only sign that persists is perilimbal redness. Mental and motor development is normal. No sensorineural or skeletal manifestations developed. Self-limited, 24h-duration, scarce urticarial-rash appeared in the age of 6 and 15 months with concomitant mild elevation of WBC/ neutrophils and SAA but normal CRP and ESR levels. Repeat MRI revealed absence of CNS involvement and lumbar puncture was not repeated. No adverse reactions presented apart from 1 URI after 12-months of canakinumab treatment.

## Conclusion

Early initiation of canakinumab-treatment in CINCA leads to disease-remission and appears to prevent the development severe disease-sequelae such as CNS, sensorineural and skeletal manifestations. The patient presented no severe adverse reactions.

## Consent to publish

Written informated consent for publication of their clinical details was obtained from the patient/parent/guardian/relative of the patient.

